# Development of a Multifunctional Bioerodible Nanocomposite Containing Metronidazole and Curcumin to Apply on L-PRF Clot to Promote Tissue Regeneration in Dentistry

**DOI:** 10.3390/biomedicines8100425

**Published:** 2020-10-16

**Authors:** Denise Murgia, Giuseppe Angellotti, Alice Conigliaro, Francesco Carfi Pavia, Fabio D’Agostino, Marco Contardi, Rodolfo Mauceri, Riccardo Alessandro, Giuseppina Campisi, Viviana De Caro

**Affiliations:** 1 Dipartimento di Discipline Chirurgiche, Oncologiche e Stomatologiche, Università degli Studi di Palermo, 90127 Palermo, Italy; giuseppe.angellotti@unipa.it (G.A.); rodolfo.mauceri@unipa.it (R.M.); giuseppina.campisi@unipa.it (G.C.); 2 Dipartimento di Scienze e Tecnologie Biologiche Chimiche e Farmaceutiche (STEBICEF), Università degli Studi di Palermo, 90123 Palermo, Italy; viviana.decaro@unipa.it; 3 Dipartimento di Biomedicina, Neuroscienze e Diagnostica avanzata Università degli Studi di Palermo, 90127 Palermo, Italy; alice.conigliaro@unipa.it (A.C.); riccardo.alessandro@unipa.it (R.A.); 4 Dipartimento di Ingegneria, Università degli Studi di Palermo, 90128 Palermo, Italy; francesco.carfipavia@unipa.it; 5 Istituto per lo Studio degli Impatti Antropici e Sostenibilità dell’Ambiente Marino, Consiglio Nazionale delle Ricerche (IAS—CNR), Campobello di Mazara, 91021 Trapani, Italy; fabio.dagostino@cnr.it; 6 Smart Materials, Istituto Italiano di Tecnologia, 16163 Genova, Italy; marco.contardi@iit.it

**Keywords:** bone regeneration, L-PRF, nanocomposite, curcumin, metronidazole, tooth extraction, nanostructured lipid carriers, hydrophilic sponge, hyaluronic acid

## Abstract

Teeth extractions are often followed by alveolar bone reabsorption, although an adequate level of bone is required for reliable rehabilitations by dental implants. Leukocyte and platelet-rich fibrin (L-PRF) has been widely applied in regenerative procedures and with antibiotic and antioxidant agents could play an essential role in hard and soft tissue healing. In this work, a nanocomposite (Sponge-C-MTR) consisting of a hyaluronate-based sponge loaded with metronidazole (MTR) and nanostructured lipid carriers containing curcumin (CUR-NLC) was designed to be wrapped in the L-PRF™ membrane in the post-extraction sockets and characterized. CUR-NLCs, obtained by homogenization followed by high-frequency sonication of the lipid mixture, showed loading capacity (5% w/w), drug recovery (95% w/w), spherical shape with an average particle size of 112.0 nm, and Zeta potential of −24 mV. Sponge-C-MTR was obtained by entrapping CUR-NLC in a hydrophilic matrix by a freeze-drying process, and physico-chemical and cytocompatibility properties were evaluated. Moreover, the aptitude of CUR and MTR to the penetrate and/or permeate both L-PRF™ and porcine buccal tissue was assessed, highlighting MTR penetration and CUR accumulation promoted by the system. The results positively support the action of nanocomposite in dental tissues regeneration when applied together with the L-PRF™.

## 1. Introduction

Teeth extractions are common practices in oral surgery, although they are often associated with several post-surgical effects such as pain, inflammation, bruising, bleeding, and infections [1]. A loss of alveolar bone may also occur as an outcome of a remodelling process, leading to significant changes in ridge dimensions, both horizontal and vertical [2,3]. This alteration takes place in the post-extraction socket during the wound healing of soft and hard tissues, and it may outwear the new bone formation process [4].

To achieve the ideal functional and aesthetic prosthetic reconstruction following the implant insertion, an adequate alveolar bone is required [5]. For this reason, several solutions to reduce the ridge reabsorption after dental extractions aimed at bone preservation or regeneration were developed [3]. The application of block grafting materials can successfully control the crestal bone reabsorption, while procedures such as lateral and transcrestal sinus floor elevation as well as guided bone regeneration (GBR) with different types of biomaterials are usually performed to improve the bone level in the absence of ridge preservation [2,6].

On the other hand, autologous plasma concentrates are a new approach for tissue regeneration in various surgical fields, such as dental and oral surgery. Specifically, they are used in the post-extraction sockets, where they seem to promote healing and soft tissue epithelialization, in procedures relating to the placement of osseointegrated implants, for the treatment of infrabony periodontal defects and periodontal plastic surgery, and, recently, they have been also investigated for bone regeneration [7,8,9].

Platelet aggregates are autologous sources of various growth factors, such as VEGF (vascular endothelial growth factor), PDGF-AB (platelet-derived growth factor AB), and TGFb-1 (transforming growth factor b-1) [7,9], which are also found during natural healing and are able to stimulate cell proliferation, matrix remodelling, and angiogenesis [8].

They are classified into four categories depending on leucocyte and fibrin content: pure platelet-rich plasma (P-PRP), leucocyte and platelet-rich plasma (L-PRP), pure platelet-rich fibrin (P-PRF), and leucocyte and platelet-rich fibrin (L-PRF) [10].

L-PRF represents the second generation of platelet aggregates. The PRF clot naturally forms a definite fibrin matrix with platelets and leucocytes entrapped in a complex three-dimensional structure without the addition of anticoagulants or gelifying agents [11]. Platelets are activated during an easy, reproducible, and low cost production process, obtaining clots that exhibit a slower dissolution than the first aggregates [12].

The treatment of alveolar bone atrophy in the post-extraction sockets is essential, but bone reabsorption and remodelling can also occur before tooth extractions due to several oral diseases, such as periodontal disease, periapical pathology, or trauma to teeth and bone [5,13]. Furthermore, since infection is a major cause for failure or delay in the bone healing process, an antibacterial could play an essential role in bone rehabilitation [14].

Metronidazole (MTR) is an antibiotic from the nitro-5-imidazole family, often used for the treatment of periodontal lesions for its effect against anaerobic organisms, strongly associated with the damage of periodontal tissue [15]. The systemic administration is associated with many adverse reactions. Most of them involve the gastrointestinal tract, producing diarrhoea, nausea, vomiting, and pseudomembranous colitis, and side effects such as hypersensitivity and gastrointestinal intolerance. Moreover, the prolonged use of MTR can increase the risk to develop a bacterial resistance [16,17]. Instead, a local treatment leads to a higher concentration of drug in the target site with a lower dosage, decreasing the adverse effects [16]. In addition, MTR seems to be a decisive surgical adjuvant of platelets aggregate during bone graft surgery. In fact, it can limit the biomaterial contamination by anaerobic bacteria, protect from infection and inflammatory reactions the early bone construction phases, and act against the possible systemic preoperative contaminations [10].

Bone reabsorption can also occur due to an overproduction of reactive oxygen species (ROS) and, consequently, oxidative stress not balanced by antioxidants. This scenario slows down the wound healing process and activates the differentiation of preosteoclasts in osteoclasts, altering the bone metabolism [18].

Curcumin (CUR) is a polyphenolic compound known for its multi-faced therapeutic actions and biological activities, such as antioxidant, anti-inflammatory, antimicrobial, anti-arthritic and chemopreventive properties [19,20,21]. This latter is also widely applied in dentistry [22,23]. After teeth extraction, the topic administration of CUR can decrease soft tissue inflammation, protect the wound from bacterial infections, increase collagen deposition and stimulate cell proliferation, improving tissue regeneration [24]. Due to its lipophilic nature and physicochemical instability [25], CUR needs to be included in a pharmaceutical formulation to be available in aqueous environments. For instance, CUR can be loaded in lipid-based colloidal dispersions, suitable formulations to overcome problems of degradation, poor solubility, and penetration inside the tissue [26,27].

Nanostructured lipid carriers (NLC) are considered the new generation of lipid nanoparticles. They are composed of a mixture of biocompatible and biodegradable liquid and solid lipids with short and long chains. NLCs can actively improve the skin penetration and the retention of drugs in the skin, and, compared to previously solid lipid nanoparticle (SLN), they show some advantage as a higher drug loading and minimized drug expulsion phenomena [26].

Recently, sponges, defined as a dispersion of gas in a solid matrix with a porous structure, have been developed as a new dosage form for the design of wound dressing and transmucosal drug delivery systems. They are prepared by removing water from a polymer matrix by a freeze-drying procedure to obtain a solid, dried, soft, and flexible structure in which NLCs could also be entrapped [28,29,30].

The aims of the present work are the development and the characterization of a nanocomposite consisting of a hyaluronate-based sponge loaded with metronidazole (MTR) and nanostructured lipid carriers containing curcumin (CUR-NLC) and obtained by a freeze-drying process. To have a suitable size for the post-extraction sockets, sponges were compressed, and their behaviour alone and together with L-PRF membranes was evaluated.

The obtained results encourage the possibility to use these sponges to deliver antioxidant and antimicrobial agents to promote ridge preservation, prevent bone reabsorption, and support bone regeneration on surgical sites of teeth extractions treated with L-PRF.

## 2. Experimental Section

### 2.1. Materials

Curcumin was purchased from Alfa Aesar (Karlsruhe, Germany), isopropyl palmitate (IP), glycyrrhetic acid (GA), sodium hyaluronate (HyNa), and metronidazole from A.C.E.F S.p.a. (Fiorenzuola d’Arda, Italia), 1-hexadecanol (HEXA), Tween 80 (Tw80), Pluronic F-68 (P-68), and polyvinylpyrrolidone (PVP-K90) from Sigma Aldrich (Saint Louis, MO, USA), and Trehalose dihydrate (TRH) from la Hayashibara Shoji (Okayama Japan). Isopropyl myristate (IM) and Polysorbate 20 were from Farmalabor, Canosa di Puglia, Italy.

Homo sapiens bone marrow stromal cells (HS5) were purchased from ATCC^®^ and were cultured in Dulbecco’s modified Eagle medium (DMEM) (ATCC^®^ 30-2002) with 10% foetal bovine serum (FBS) and antibiotics at 37 °C in a humidified incubator under 5% CO 2 and 95% air.

Non-enzymatic artificial plasma pH 7.4 (PBS) was prepared dissolving 2.80 g of KH_2_PO_4_ and 20.5 g of Na_2_HPO_4_ in1 L of distilled water, and the same conditions were used for non-enzymatic artificial plasma pH 7.4 (PBS) with 4% of Polysorbate 20.

Buffer pH 6.8 solution simulating saliva was prepared using NaCl (0.126 g), KCl (0.937 g), NaHCO_3_ (0.631 g), KSCN (0.655 g), KH_2_PO_4_ (0.2 g), Urea (0.2 g), Na_2_SO_4_ (0.154 g), NH_4_Cl (0.178 g), CaCl_2_ (0.130 g), and NaHCO_3_ (0.631 g) in distilled water; 0.9% saline solution was prepared by dissolving 9 g of NaCl in 1 L of distilled water.

Citrate buffer 10 mM (pH 6.2) used for NLC preparation was obtained dissolving 2.675 g of sodium citrate dehydrate and 0.190 g of citric acid monohydrate in 1 L of distilled water. All chemicals and solvents of analytical grade were purchased from VWR International (Milano, Italy) and used without further purification. Porcine mucosae were kindly supplied by the Municipal Slaughterhouse of Villabate (Palermo, Italy).

For data processing, Kaleidagraph v. 3.5 (Synergy Software Inc, Reading, PA, USA) and Curve Expert 1.34 (DG Hyams, Starkville, MS, USA) software for Windows were used.

### 2.2. Evaluation of CUR Solubility in Lipid Mixtures

Our previous studies showed that HEXA, IP, and GA were suitable for forming NLCs [28]. An amount of 50 or 80 mg of CUR was added to 950 or 920 mg of lipophilic mixture of HEXA, IP, and GA mixed in various ratios (Table 1) and pre-heated at 70 °C under stirring by a silicon bath on a hot plate (Heidolph MR2001 K equipped with EKT 3001 electronic temperature control, Germany) and were maintained in these conditions for one hour. The absence of drug crystals in the clear melted lipid mixture indicated the solubility of the drug in the lipids tested. The analysis was carried out in triplicate. Then, the lipid mixtures considered good were cooled at room temperature and finally stored at 2–4 °C until the subsequent use.

### 2.3. Preparation of CUR-Loaded NLC

For the CUR loaded NLCs, MIX7 was chosen, and samples were prepared by homogenization followed by probe sonication method. Briefly, 20 mL of 10 mM citrate buffer pH 6.2 containing 1% (w/v) Tween 80 (Tw80) and/or 1% (w/v) Pluronic F-68 (F-68), previously brought to 90 °C, was added to the melted lipid mixture (100, 200, or 300 mg) and firstly homogenized at 30,000 rpm (Polytron Model PT MR 2100, Kinematica, Switzerland) for 1 min and then sonicated by an ultrasonic homogenizer SONOPULS (Bandelin, mod. HD 2070) for 10 min in pulsatile mode with cycles of 0.7 s of activity and 0.3 s of inactivity. An additional ultrasonic treatment of 10 min, placing the container in an ice bath, was then carried out. The drug-free nanoparticles were also prepared. The compositions of the prepared NLCs are given in Table 2.

### 2.4. Preparation of CUR-Loaded NLC Dispersion Enriched in Metronidazole

M7-300-TP dispersion was chosen for the next step. In order to obtain a homogeneous distribution of metronidazole (MTR) in the M7-300-TP dispersion, 150 mg of MTR were solubilized in 20 mL of hot 10 mM citrate buffer pH 6.2 containing the surfactants before the CUR-NLC preparation. Therefore, the CUR-NLC with MTR dispersion was prepared in the same way as previously described, and the dearth of drug crystals was always checked by an optical microscope.

### 2.5. Characterization of Nanostructured Lipid Carriers

#### 2.5.1. Particle Size, Polydispersity Index, and Zeta Potential Analysis

The mean particle size, the polydispersity index (PI), and the zeta potential (ZP) were determined by dynamic light scattering (DLS) using a Nano-ZS Zetasizer (Malvern Instruments, Worcestershire, UK). DLS is a technique for measuring the fluctuations in scattered light intensity due to diffusing particles. To avoid multiple scatterings of the light caused by a high concentration of particles, the NLC dispersions were diluted with purified water at an appropriate ratio from 1:99 to 1:999. All experiments were performed at 25 °C under an electrical field of 40 V7 cm for ZP analyses on the day NLCs were prepared. The results of all NLCs analysed are reported in Table 2.

Results of particle size, PI, and ZP were presented as the mean value standard deviation (SD) of six runs.

#### 2.5.2. Morphology by Scanning Electron Microscopy (SEM) and Transmission Electron Microscopy (TEM)

To evaluate the morphology and the topographic characteristics of both CURNLC-MTR and CUR-NLC dispersions, SEM analyses were performed.

Measurements were carried out using a scanning electron microscope (Zeiss EVO MA10, Oberkochen Germany) equipped with an SE-Everhart-Thornley secondary electron detector with lanthanum hexaboride (LaB_6_) cathode as the source of electrons, an accelerating voltage of 20 keV and probe voltage of 10 pA.

The scanning electron microphotographs were acquired in high-vacuum condition (HV, about 10^−7^ mbar) and magnified up to ×50.000 (200 nm).

To increase the surface electrical conductivity, for the CUR-NLCs analysis, a few drops of fresh CUR-NLC dispersion were put on an aluminium stub, placed in a CaCl_2_ desiccator at 8 °C for 24 h, and then coated with an ultrathin layer of gold (thickness about 2 nm) with an AGAR Sputter Coater type system. For the samples of sponges loaded with MTR and CUR-NLC, a gold coating was applied before each analysis.

For the TEM measurements, one drop of diluted CUR-NLCs and CUR-MTR-NCLs (1 mg of the matrix for mL) was placed on a carbon-coated 200 mesh copper grids for 5 min and negatively stained with 1% uranyl acetate solution before being allowed to dry, and the samples were visualized by using a JEOL JEM-1011 microscope (JEOL Ltd., Tokyo, Japan) equipped with a tungsten thermionic gun operating at 100 kV accelerating voltage. The TEM images were acquired with an 11 MPx Orius 1000 CCD camera (Gatan, Pleasanton, CA, USA).

#### 2.5.3. Entrapment Efficacy (EE) of NLC

Preliminarily, the total amounts of CUR and MTR in the fresh NLC dispersion were determined by UV-Vis measuring of 100 μL of NLC dispersion diluted in 20 mL volumetric flasks brought to volume with methanol. This allowed us to determine the drug recovery (DR), calculated by the following Equation (1):

(1) $$DR\%\left(\frac{W}{W}\right)=\;\frac{\mathrm{Drug}\;\mathrm{in}\;\mathrm{formulation}\;}{\mathrm{Drug}\;\mathrm{used}\;}\times 100$$

Then, entrapment efficacy (EE) and loading capacity (LC) of NLCs were indirectly determined by quantification of free CUR (unentrapped) in the aqueous medium, applying respectively Equations (2) and (3) [31].

(2) $$EE\%\left(\frac{W}{W}\right)\;=\;\frac{\mathrm{CUR}\;\mathrm{in}\;\mathrm{formulation}-\;\mathrm{free}\;\mathrm{CUR}\;}{\mathrm{CUR}\;\mathrm{in}\;\mathrm{formulation}\;}\times 100$$

(3) $$LC\%\left(\frac{W}{W}\right)\;=\;\frac{\mathrm{CUR}\;\mathrm{in}\;\mathrm{formulation}\;}{\mathrm{Lipids}\;\mathrm{amount}}\times 100$$

Two methods were chosen to evaluate the concentration of the free drug (unentrapped) in the aqueous medium:

(a) Dialysis assay: dialysis tube (molecular weight cut off, MWCO, 12–14000 Daltons, Visking Dialysis Membrane—Medicell Membranes Ltd., London, UK) was pre-activated, filled by 1 mL of NLC dispersion, and submerged in 350 mL of distilled water, keeping it protected from the light at room temperature and under gentle magnetic stirring. After 24 h, both the dispersion inside the tube and the external aqueous phase were analysed by UV-Vis analysis.
(b) Ultrafiltration assay: 0.45 mL of fresh NLC dispersion were placed in the upper chamber of a centrifugal filter tube (Ultrafree-MC, Millipore, cut-off 10,000 NMWL, and 30,000 NMWL) and then centrifuged (Microfuge 22R, Beckman coulter™, Brea, CA, USA) at 8000 rpm at 4 °C for 30 min. The aqueous solution collected at the bottom of the tube was subjected to UV-Vis analysis to determine the free CUR content [32].

### 2.6. Preparation of Sponges Loaded with Metronidazole and Curcumin Nanostructured Lipid Carriers

In order to obtain a complete dispersion of the matrix components in the aqueous medium, two gels differing in composition were prepared and kept overnight at 4 °C. The compositions of the gels were HyNa 2.50 g, PVP-K90 0.40 g, and TRH 15.0 g in 100 mL of water and HyNa 4.0 g, PVP-K90 0.60 g, and TRH 10.0 g in 100 mL of water, respectively. An aliquot of 2 or 4 g of gel was added to the M7-300-TP or the M7-300-TP-MTR fresh dispersion, and the whole mixture was ultrasonically homogenized for 10 min with cycles of 0.7 s of activity and 0.3 s of inactivity by placing the container in an ice bath. The sample was transferred in a 50 mL falcon, frozen at −80 °C for 24 h, and then moved to the freeze-drier (Labconco FreeZone^®^ Kansas City, MO, USA 2.5 Liter Freeze Dry System), setting the vacuum at 0.014 mPa and −54 °C of temperature and maintaining these conditions for 48 h. The nanocomposite sponge compositions are reported in results and discussion section.

### 2.7. Quantitative Determination of CUR and MTR in the Sponges

A total of 5 mg of the lyophilized sponge was placed in a 20 mL flask, brought to volume with methanol, and sonicated until complete dissolution. Then, 1 mL aliquots were centrifuged (15,000 rpm for 2 min), and the supernatant was spectrophotometrically analysed to quantify CUR and MTR using the appropriate blank and the calibration curve. The analysis was carried out on three aliquots from each lyophilized sponge.

### 2.8. Hygroscopic Measurement of Sponges

The ability of sponges to attract water molecules from the atmospheric moisture at room temperature was evaluated using an analytical five decimal balance (mod. AE 240, Mettler-Toledo S.p.A., Milan, Italy). Before each test, a desiccant box containing silica, previously dried, was kept in the weighing chamber for 15 min.

Immediately after exiting the freeze dryer, an aliquot of sponge was weighed, and the two side doors of balance were opened, allowing the sponge to contact the environmental moisture. At intervals of 10 min, the weight of the sponge was recorded, taking care to reopen the balance’s doors to equilibrate sponges with atmospheric moisture. The experiment was prolonged until the powder showed no increase in weight.

The increase in weight of each sample was calculated as follows (4):

(4) $$\mathrm{Weight}\;\mathrm{increase}\;\%\;=\;\frac{W_{t}-W_{i}}{W_{i}}\;\times \;100$$

where *W_t_* is the weight of sponge at time t, and *W_i_* is the weight of dry sponge. The experiments were performed in triplicate.

### 2.9. Differential Scanning Calorimetry (DSC) Analysis

Differential calorimetric analysis was performed by a SETARAM DSC 131 EVO instrument on samples weighted of approximately 8 mg placed in aluminium capsules (Perkin Elmer). Analyses were performed using a temperature ramp from −10 °C to about 250 °C at 10 °C/min under nitrogen flow.

Samples of CUR, lipid mixture MIX 7, physical mixture of hydrophilic components (containing HyNa, TRH, PVP-K90, Tw80 and P-68), lyophilized gel (containing HyNa, TRH, PVP-K90, Tw80 and P-68), and Sponge-C were analysed.

### 2.10. Sponges Porosity Determination

The freeze-drying process allows removing the solvent from the formulation to obtain a porous structure with air in the space previously occupied by the solvent. The sponge porosity, meant as total pore volume, was calculated mathematically according the following Equation (5) [33]:

(5) $$\%Porosity=\;\frac{practical\;volume-theoretical\;volume}{pratical\;volume}\times 100$$

Theoretical volume was considered the bulk volume of the total ingredients of one sample and was calculated according to the Equation (6):

(6) $$Theoretical\;volume=\sum \frac{m_{n}}{\rho_{n}}\;$$

where m and ρ are respectively mass and density of each ingredient.

The practical volume of sponge was determined by placing the sample in a graduated cylinder and measuring the occupied volume (length × width × height).

The determinations of practical volume of sponges were performed in triplicate. Results were expressed as means ± SE.

### 2.11. ABTS Free Radical Cation Scavenging Assay on Sponges

ABTS (2,2′-azino-bis (3-ethylbenzothiazoline-6-sulfonic acid) free radical cation scavenging assay was performed as described in Contardi et al. [34]. ABTS radical cation (ABTS·+) was generated by the reaction between 7 mM ABTS water solution with 2.45 mM potassium persulfate solution in the dark at room temperature for 12–16 h. The ABTS·+ solution was diluted with water to obtain an absorbance of 1.0 a.u. at 734 nm. Afterward, Sponge-C or Sponge-C-MTR were added to 3 mL of diluted ABTS·+ solution. Specifically, 10, 7.5, 5, 2.5, and 1 mg of the two systems were used to perform the assay. Finally, 10 mg of sponge containing NLC empty (without any drugs) were also tested as a control. The decrease in absorbance was determined at 734 nm with a Cary 300 Scan UV-visible spectrophotometer at different times. All the measurements were performed in triplicate, and the results were averaged to obtain a mean value. Radical scavenging activity (RSA) was expressed as the inhibition percent of free radicals of the sample and calculated by using the Equation (7):

(7) $$Radical\;Scavenging\;Activity\;\left(\%\right)=\;\frac{A_{0}-\;A_{1}}{A_{0}}\times 100$$

where A_0_ is the absorbance value of the control radical caption solution, and A_1_ is the absorbance value of the sample at different time points.

### 2.12. Preparation of Minitablets

To standardize the sponge amount for the next analyses, minitablets were produced by compressing a precisely weighed amount (30 mg) of Sponge-C-MTR. A graduated mould having 5 mm diameter as well as a mobile upper and a lower plunger was used. A minitablet with a volume of 0.76 cm^3^ was obtained by moving the upper plunger downwards.

The diameter and the thickness of each sample were evaluated by a digital micrometre (Vogel Germany) to value the reproducibility of the preparation method used.

### 2.13. Swelling Test

Weight and optic assessments were used to perform the swelling tests on the minitablets. The weight tests were carried out, placing the minitablets on microscope slides and weighting by an analytical balance (Mettler, Columbus, OH, USA, Mod. AE 240). A total of 0.5 mL of artificial saliva (pH 6.8) were added on the tables every 5 min for 20 min. At each timepoint, the excess water was removed with a filter paper, and the weight was assessed. The water uptake was quantified gravimetrically until weight plateau or cleavage of the tablets. Tests were performed on six different minitablets, and results were reported as means ±SE (*n* = 9; *p* < 0.05) following the Equation (8).

(8) $$\mathrm{Swelling}\;\mathrm{Index}\;=\;\frac{W_{0}\;+\;\left(\mathrm{Wt}\;-{\;W}_{0}\right)}{W_{0}}\;=\;\frac{W_{t}}{W_{0}}$$

where Wt is the weight of the minitablets at time t, and W_0_ is the weight of the dry sample.

The optical assays were performed with both the plan and the frontal assessments. Samples were previously placed on a microscope slide and then on graph paper. Every 5 min for 2 h, 0.5 mL of simulated saliva (pH6.8, 37 °C) were added, and photographs were taken to evaluate any change on the minitablets’ morphology.

### 2.14. In Vitro CUR and MTR Release Studies

The ability to release CUR and MTR from the minitablets was evaluated by the in vitro release studies, carried out using the Franz diffusion cell (Permeagear, flat flange joint, 9 mm orifice diameter, 15 mL acceptor volume, SES GmbH-Analysesysteme, Bechenheim, Germany) with the minitablets (30 mg, area = 0.2 cm^2^) placed in the donator chamber and soaked with 0.5 mL of citrate buffer (pH 6.2) to allow the swelling. A cellulose nitrate membrane (Millipore) with a 0.45 µm cut-off, previously soaked with the acceptor fluid, was fixed between donator and receiver compartments [35]. To value the CUR release, the acceptor chamber was filled with isopropyl myristate, while for MTR, phosphate buffer pH 6.2 plus tween 20 (4% w/v) was used to increase the drug solubility in the media [36]; in both cases, the temperature was kept at 37 ± 0.5 °C for the whole experiment.

The samples (0.5 mL) were withdrawn from the receiver compartment at time points of 15 min, replacing the same volume with fresh fluid. All the CUR withdraws were diluted 1:1 with CH_2_Cl_2_, then all the samples were analysed by UV-Vis analysis using the appropriate blank and calibration curve. The experiments were carried out for 3 h, results were reported as means ± SE (*n* = 6), and all the release data were elaborated by Kaleidagraph v.3.5 software and fitted to the semi-empirical equation usually applied to describe drug release from polymeric systems [37]. Fittings were validated by using χ2 where a *p*-value of less than 0.05 was considered statistically significant.

### 2.15. Ex Vivo Permeation/Penetration Studies of CUR and MTR from Minitablets through L-PRF Membrane

The L-PRF membranes were prepared following the IntraLock IntraSock ™ protocol, approved by the Food and Drug Administration (FDA).

The patient’s blood was collected in suitable IntraSpin tubes (8 mL) and centrifuged using the calibrated and tested IntraSpin centrifuge at 2700 rpm for 12 min to ensure a proper blood separation and the L-PRF formation. The obtained matrix was separated from the fluid component of the blood and carefully laid down on the Xpression Fabrication Kit, expressly designed to separate the serum from the fibrin clot in a controlled manner and to form one thin and compact layer of L-PRF.

The L-PRF membranes were repeatedly washed with a saline solution to remove any blood residues, and then they were fixed between the two compartments of the jacketed Franz type diffusion cells to perform the ex vivo permeation/penetration studies of CUR and MTR from minitablets.

The donor chamber was filled by the minitablet and 0.5 mL of citrate buffer pH 6.2, while the citrate buffer pH 6.2 plus 4% (w/v) of Tween 20 was used as receiver fluids. The experiments were carried out for 3 h, withdrawing 0.5 mL every 15 min, replacing the same volume with fresh fluid, and keeping the system temperature at 37 ± 0.5 °C. At the end of each experiment, the amounts of CUR and MTR entrapped inside the L-PRF membrane were quantified by the extraction in methanol. Each L-PRF membrane used in permeation experiments was washed with PBS (3 × 2 mL) and dipped for 5 min in warmed (50–60 °C) methanol (3 mL). The extraction was repeated three times. The extracted liquors were collected, transferred in a 10 mL flask, and brought to volume with methanol. The amount of CUR extracted was quantified by UV-Vis, as described above. The same procedure was carried out also on L-PRF membranes subjected to the experimental phase in the absence of sponge and used as a control. Data were reported as the percentage amount of CUR and MTR into the tissue.

### 2.16. Ex Vivo Permeation/Penetration Studies of CUR and MTR from Minitablets and Sponges through Porcine Buccal Mucosae

Mucosal specimens consisted of tissue removed from the inner cheek (buccal area) of freshly slaughtered domestic pigs. After sampling, all specimens were immediately placed in PBS (pH 7.4), transferred to the laboratory in a refrigerated transport box within 1 h, and used within 2 h from animal sacrifice. Excesses of connective and adipose tissue were trimmed away. Specimens were dipped for approximately 1 min in a pre-warmed (60 °C) saline solution; the connective tissue was then carefully peeled off from the mucosa (slides 250 ± 25 mm thick) to obtain the heat-separated epithelium along with the intact basal lamina [38]. The thickness was measured using a digital micrometre. Then, specimens were equilibrated in PBS for about 3 h at room temperature to remove biological matter, which could interfere with drug analyses. The equilibration medium was replaced with fresh PBS every 15 min. Appropriate sections of mucosa were mounted in vertical jacketed, Franz type diffusion cells (Permeagear, flat flange joint, 9 mm orifice diameter, 15 mL acceptor volume, SES GmbH—Analysesysteme, Bechenheim, Germany), used as a two-compartment open model.

The acceptor compartment was filled with citrate buffer pH 6.2 containing 4% Tween^®^ 20 and, again, the tissue disks (12 mm diameter) were equilibrated at 37 ± 0.1 °C adding 0.5 mL of citrate buffer pH 6.2 in the donor compartment. After 1 h, the donator fluid was replaced with the minitablet and 0.4 mL of simulated saliva pH 6.8. At an interval of 30 min for 6 h, 0.5 mL of the acceptor fluid was withdrawn and replaced with fresh fluid; the temperature was kept at 37 ± 0.5 °C for the whole experiment. The amount of drugs permeated through the porcine buccal mucosa was spectrophotometrically determined. The residual drugs entrapped into the mucosal tissue was extracted at the end of each ex vivo experiment following the above-mentioned method and was quantified by the UV-Vis spectrophotometry.

The same experiment was performed investigating the amount of CUR entrapped in the mucosae specimens versus time, stopping each one at 0.5, 1, 1.5, 2.5, 3.5, 5, and 6 h. At the end of each experiment, the porcine tissue was extracted as described above and CUR quantified by UV-Vis spectrophotometry. Experiments were performed in triplicate.

### 2.17. MTT Cell Viability Assay

Homo sapiens bone marrow stromal cells were grown in DMEM (ATCC^®^ 30-2002) with 10% foetal bovine serum (FBS) and antibiotics and maintained in culture in a humidified incubator under 5% CO_2_ and 95% air at 37 °C.

To assay cell viability effects on HS5, cells were seeded at 30,000 cells/cm^2^. The day after, the medium was removed and substituted with fresh medium containing different concentrations of the empty nanocomposite sponge (without CUR and MTR) or Sponge-C-MTR. The following concentrations were tested: 0.025 mg/mL, 0.0502 mg/mL, 0.080 mg/mL, 0.12 mg/mL, and 0.15 mg/mL.

MTT assay (Invitrogen M6494), which measures the metabolic reduction of 3-(4, 5-dimethylthiazol-2yl)-2, 5, diphenyl tetrazolium bromide to a coloured formazan by viable cell, was used to evaluate cell viability at 24 h after the treatment. Experiments were performed in triplicate.

### 2.18. Drugs Quantification by UV-Vis Methods

The amounts of CUR and MTR entrapped in the NLC suspension, the sponges, and the membranes were measured spectrophotometrically (UV-Vis mod. Pharma Spec 1700, Shimadzu, Tokyo, Japan) using the appropriate calibration curve and blank. The linearity was statistically determined by regression analysis of five concentrations in the range below specified and evaluated in triplicate. The accuracy was determined by adding known concentrations of the drug in the solutions followed by their UV analysis by relative method. Three different concentrations in triplicate covering the studied range were selected and analysed for the recovery. The precision of the developed methods was determined by calculating relative standard deviation (%RSD) of the mean recoveries. UV-Vis methods were found as simple, accurate, and reproducible.

For CUR determination, two calibration curves in methanol were performed at λmax = 428 nm; in the linearity range of 0.0002–0.0075 mg/mL, regression equation was Abs = −0.0289 + 132 × [mg/mL] (R = 0.999, SE 0.0101), and in the linearity range of 0.0025–0.01 mg/mL, regression equation was Abs = −0.0108 + 136 × [mg/mL] (R = 0.997, SE 0.0397).

For the calibration curve in phosphate buffer pH 6.2 plus tween 20 (4% w/v) in the linearity range of 0.0001–0.001 mg/mL, the regression equation was Abs = 0.01277 + 160 × [mg/mL] (R = 0.997, SE 0.0105).

For CUR release experiments, the calibration curve in dichloromethane:isopropil miristate (50:50) was in the linearity range of 2.5 × 10^−5^ mg/mL−5 × 10^−4^ mg/mL with the regression equation Abs = 1.40 × 10^−3^ + 1.17 × 10^2^ × [mg/mL] (R = 0.998, SE 0.0015)

For MTR determination, validation parameters in methanol, in PBS, and in phosphate buffer pH 6.2 plus tween 20 (8% w/v) were found at λ_max_ = 310 nm. For PBS, linearity range was 0.0025–0.0125 mg/mL, and the regression equation was Abs = 0.0011 + 61.78 × [mg/mL] (R = 0.999, SE 0.0040). For phosphate buffer pH 6.2 plus tween 20 (8% w/v), linearity range was 0.0025–0.02 mg/mL, and regression equation was Abs = 0.0771+ 46.8 x [mg/mL] (R = 0.998, SE 0.0102). Finally, for methanol, linearity range was 0.0025–0.03 mg/mL, and regression equation was Abs = 0.0011+ 62 × [mg/mL] (R = 0.999, SE 0.0092),

No interferences between drugs and components of formulations were observed at the testing concentrations, and no change in drug absorbance at its λmax was experienced in the presence of excipients. The amount of drugs founded in acceptor and/or entrapped in the membrane was measured after withdrawal or extraction from mucosal tissue by methanol. Intraday and interday variations observed during the collection of experimental data were lower than sensibility.

### 2.19. Data Analysis

Data were expressed as mean ± SE of at least three replicates (*n* = 3). For the statistical analysis of data, Student’s *t*-test was applied with the minimum levels of significance with *p* < 0.05. One-way ANOVA followed by Dunnett’s multiple comparison tests were done for the biological characterization by the use of GraphPad Prism 5 (GraphPad Software, San Diego, CA, USA).

## 3. Results

CUR possesses extremely low aqueous solubility in both acidic and neutral pHs, with a reported value of 11 ng/mL in aqueous pH 5.0 buffer solution, while it is soluble in ethanol, methanol, and acetone [39]. The rapid CUR decomposition in neutral-basic pH conditions is also well known [40]. On the other hand, CUR is an excellent candidate for the development of formulations using nanoparticle lipid systems [41] due to its poor solubility and high instability in aqueous fluids.

In the pharmaceutical field, nano-encapsulation is highly exploited to promote the absorption of substances which, as such, would not cross biological membranes, using natural and/or synthetic materials to obtain formulations suitable for CUR administration. The physicochemical characteristics of lipid materials, such as polymorphism, lipid miscibility, and stability, and the surfactant effect on structure and type of nanoparticles are the most relevant parameters to take into account in formulation [42]. In addition, among all the lipid materials, those that do not undergo light or heat degradation or oxidation processes must be chosen. From the biological point of view, the excipients used for the synthesis of NLCs must be non-carcinogenic or teratogenic, non-allergenic, and removed from the body through the physiological metabolic processes. Since the biocompatibility of all the components with the biological tissues is necessary, and considering the lipid nature of the active ingredient, our choice fell on 1-Hexadecanol (HEXA), isopropil palmitate (IP), and 18-β glycyrrhetic acid (GA).

HEXA is a long-chain fatty alcohol widely used in cosmetic products with emollient properties and weak antimicrobial activity [43]. IP is a branched and low molecular weight liquid ester with emollient characteristics that is easily absorbed and has a suitable solvent capacity for lipophilic active ingredients. GA is an active triterpenoid metabolite of glycyrrhizin extracted from the dried roots and rhizomes of *Glycyrrhiza glabra*, existing as α and β stereo-isomeric forms [44]. This well known compound shows beneficial effects as skin whitening and anti-inflammatory agents in acute and chronic dermatitis, reducing skin lesion sizes and antioxidant activity against b-carotene destruction and LDL) oxidation [45]. Furthermore, antiviral, antibacterial, antifungal, antitussive, anti-diabetic, anti-diuretic, anti-ulcer, antihepatotoxic, cytoprotective, and cytotoxic effects were also demonstrated [46,47,48].

To prepare an NLC with high CUR loading and excellent stability, it is crucial for there to be complete solubilization of CUR in the used lipid mixture. Therefore, it was necessary to investigate CUR solubility in the lipophilic mixture by varying the ratio of lipid components. The solubility of CUR in an amount corresponding to 5 and 8% w/w of the whole mixture was tested, modifying, in turn, lipid components ratios. The screening results obtained by visual inspection upon heating showed that none of the lipid mixtures in the tested ratios were able to solubilize CUR in an amount corresponding to 8% (w/w) of the whole mix. On the contrary, 5% CUR was completely solubilized when GA was present at 3%, and HEXA and IP were in 60:40 or 50:50 ratios (Table 1).

For the CUR loaded NLCs, MIX7 was chosen, and CUR-NLCs were prepared by homogenization followed by probe sonication method. To avoid the pH-dependent CUR decomposition [49], the pH 6.2 citrate buffer was chosen as aqueous medium. All the NLC dispersions obtained by changing both the lipid mixture ratios and the surfactants (Table 2) were homogeneous, stable, and easy to re-disperse.

DLS measurements of nanoparticle mean particle size (Z-average), particle size distribution (PDI), and electrical charge surface (Z-potential, ζ) were analysed to characterize the NLC dispersions and choose the one with the best parameters, since these factors are usually investigated to value the colloidal system stability.

Acceptable NLC dispersions presented PDI values between 0.1–0.4, indicating that the lipid nanoparticles were monodispersed, with narrow population sizes, low variability, and no aggregation. NLCs with Z-potential value close to ±30 mV are considered stable because the electrostatic repulsion between particles with the same electrical charge prevents aggregation. Table 3 shows Z-average, PDI, and Z-potential values of all the studied formulations.

The combined use of Tween 80 and Pluronic F-68 as surfactants had a high influence on the results, especially on PDI values, since less width of the particle size distribution was observed. Due to a synergic action between the two emulsifying agents, mixed films formed on nanoparticle interfaces, leading to better surface coverage and increased viscosity and enhancing the long term stability of the formulation [42,50].

According to these conditions, M7-300-TP showed the most suitable values for all the studied parameters and was chosen to prepare subsequent formulations.

Since the aim of the work was to obtain a bifunctional nanocomposite also loaded with the antibacterial agent MTR, its physicochemical characteristics needed to be considered. MTR shows high solubility in water (10.61 mg/mL), particularly at a low pH, having pKa (basic) of 2.49 and LogP = −0.02 [51,52].

Therefore, it is conceivable that MTR could remain entrapped in the hydrophilic portion of the composite drug delivery system designed by us. However, its solubilization rate is low, and the risk of MTR not being wholly solubilized in the next step of sponge formation could be high. Therefore, to be sure that the whole MTR dose (150 mg) was solubilized in the aqueous phase of the dispersion, we decided to add MTR in the first step consisting of NLCs preparation, in particular to M7-300-TP formulation, obtaining the sample M7-300-TP-MTR. The formulation was characterized and compared in terms of Z-average, PDI, and Z-potential to the others in order to evaluate MTR effects on these parameters (Table 3).

Both average particle size and PDI values decreased when MTR was added to the formulation, while Z-potential values were not significantly affected by it.

**Figure 1:**
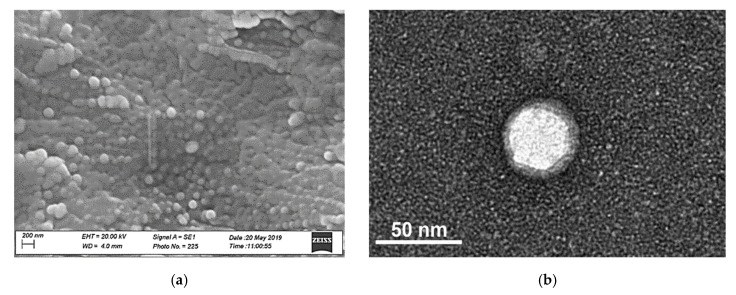
(**a**): SEM morphology of dried dispersion M7-300-TP-MTR; bar = 200 nm. (**b**): TEM image of single NLC from M7-300-TP-MTR batch; bar = 50 nm.

The M7-300-TP-MTR sample was chosen for the next characterizations. In order to confirm the shape and the size of NLCs as well as to investigate their surface morphology, the electron microscopy scan (SEM) was performed on M7-300-TP-MTR, and the micrographs are shown in Figure 1a. The nanoparticles appeared with spherical and smooth shapes, and the sizes were less than 200 nm, confirming the DLS analysis with homogeneous dimensional distribution.

No crystalline structures of MTR were observed, suggesting the absence of drug crystals in the dispersions. The particle agglomerations detected may have been due to the sticky nature of lipids as well as the treatment performed on the sample prior to the SEM analysis.

In Figure 1b are reported TEM images of single NLCs loaded only with CUR and CUR-MTR. As can be noticed, the detail of the single NLCs shows an average size of 50 ± 10 nm and well defined circular structure.

### 3.1. NLCs Characterization

Preliminarily, the amount of CUR and MTR in the fresh NLC dispersion, expressed in terms of DR, was determined to evaluate the amount of active and inactive ingredients lost during the preparation step. CUR and MTR recovery (DR) was calculated using the effective volume of the fresh NLCs dispersion recovered, resulting in 93 ± 1.63% and 88 ± 2.04% (mean ± SE), respectively. Entrapment efficacy (EE%) and loading capacity (LC%) of NLCs were indirectly determined by quantification of the free CUR in the aqueous phase (unentrapped) by both dialysis and ultrafiltration methods. Since no free CUR was detected by both the analyses, the EE and the LC resulted respectively in 100% and 5% of recovered materials. These results confirm the high affinity of CUR towards lipid mixtures and optimal experimental conditions to produce NLCs.

Regarding MTR, by the analyses, up to 94% of the drug was unentrapped, demonstrating that, during the NLCs synthesis, MTR remained in aqueous medium, and about 5% could be partitioned among the NLCs. However, this did not alter the NLCs characteristics.

### 3.2. Sponges Loaded with MTR and CUR-NLCs Formulation and Characterization

Sponges were obtained by drying M7-300-TP or M7-300-TP-MTR dispersions entrapped in a bioerodible hydrophilic polymers matrix. The designed matrix should be capable of adhering to the tissues of the post-extraction sockets and swell once soaked by the physiological fluids, allowing the release of the antibiotic and the NLC containing the antioxidant agent. This formulation was designed following two aims: (1) to allow the tissue penetration of CUR, otherwise insoluble in biological fluids, by its conveying into lipid nanoparticles; (2) to promote MTR prolonged loco-regional release in the post-extractive socket to preserve aseptic conditions of the target. To achieve this purpose, suitable hydrophilic polymer matrices were formulated. A mixture of polymers, such as hyaluronic acid and PVP, was chosen to prepare a three-dimensional network to entrap NLC dispersion and obtain a solid composite such as a sponge by freeze-drying method. Hyaluronic acid (HA) was selected as biocompatible and biodegradable polymer, used in the dental field as wound healing and adjuvant in tissue regeneration [53]. Polyvinylpyrrolidone (PVP-K90) is one of the most utilized pharmaceutical polymers for its chemical and biological inertia. It has been shown to have an excellent affinity with mucins and a capability of forming solid matrix systems (e.g., films, tablets, discs) with enhanced mucoadhesive properties [54,55,56]. In addition, Trehalose (α-D-glucopyranosyl-α-D-glucopyranoside, TRH) a hydrophilic and biocompatible sugar as a cryoprotective agent, was added. Due to the ability to establish hydrogen bonds with bio-structures, TRH is able to prevent the degradation of biological materials replacing water or interposing itself between water and them [57].

The multicomposite sponges were prepared by adding 2 g or 4 g of gels to M7-300-TP or M7-300-TP-MTR dispersion followed by homogenization and freeze-drying treatments. Sponges compositions obtained are shown in Table 4.

All the samples appeared solid, friable, and uniform in colour after drying, but an alteration of Sponge-A and Sponge-A-MTR occurred once in contact with the environmental temperature and moisture. In fact, when they were taken out of the freeze dryer, they became brown and sticky, also showing hygroscopicity. This behaviour has been attributed to the too low amount of polymers, which did not allow it to maintain separation of the lipid nanoparticles, leading to the mutual blending of them.

The amount of the active compounds was evaluated by analysing different portions of powder of the same batch and quantifying the drugs by UV-Vis analysis. CUR and MTR resulted in 0.9 ± 0.03% and 9.5 ± 0.02% w/w, respectively, showing a homogeneous distribution of active compounds in the whole samples.

**Figure 2:**
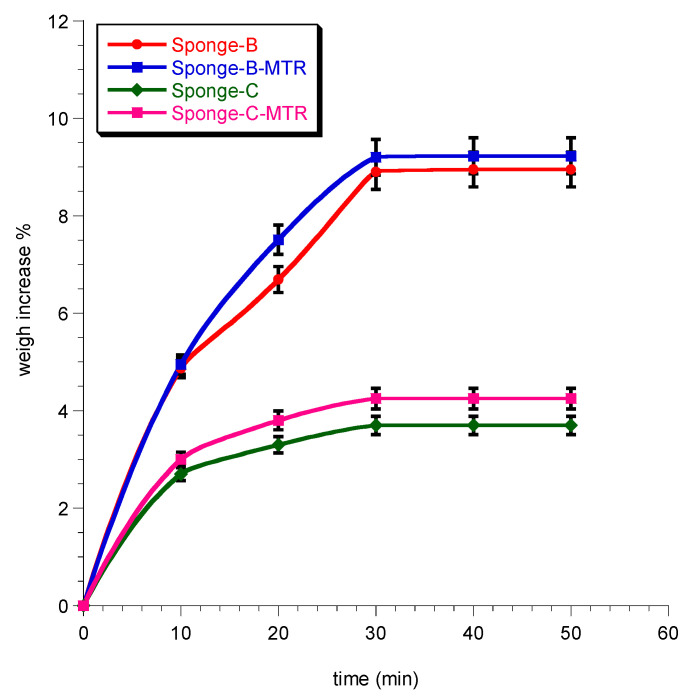
Percent of weight increase of sponges maintained in environmental moisture over time. Data represent mean ± SE (*n* = 3).

The ability of the sponges to take up moisture from their surroundings was evaluated as weight increase over time. The solid-state of the samples was ensured during the experiment to exclude potential transformations once the moisture was absorbed. Figure 2 reports the gradual increase of the weight up to 30 min. At this time, a plateau was observed, suggesting that the equilibrium with environmental moisture was reached.

DSC analyses were carried out to evaluate the amorphous or the crystalline state of each component as well as the stability of the formulation or the presence of any polymorphisms.

**Figure 3:**
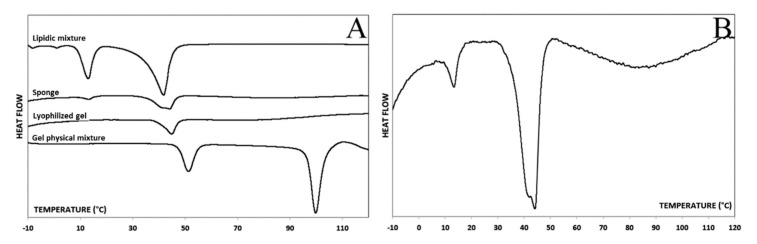
(**A**) Thermograms of the lipid mixture, Sponge-C, lyophilized gel, and gel physical mixture; (**B**) Thermogram of the Sponge-C with higher magnification.

Figure 3A shows the thermograms of the different analysed samples. In the thermogram of the physical mixture of gel components, the melting peak of TRH (the component with the higher concentration in the sample) was clearly visible. Moreover, in the same thermogram, the melting peak of P-68, characterized by a melting temperature of 52 °C, was clearly discernible. In the thermogram obtained analysing the sample after the freeze-drying, it was easy to notice the effects of the process. A partial amorphization of P-68 occurred, witnessed by a decrease of melting enthalpy (from 37 to 20 J/g) and melting temperature (from 52 to 45 °C). Moreover, the melting peak of the TRH was not detected, leading one to speculate about its complete amorphization during the process.

The thermogram concerning the lipid mixture prepared 24 h before the analysis presented two melting peaks at 16 °C and 40 °C, corresponding to IP and HEXA, respectively.

The thermogram of the Sponge-C (which, for the sake of clarity, is also shown in Figure 3B at higher magnification) shows the IP melting peak as well as a composite melting peak between 44 and 46 °C, corresponding to a merge of P-68 and HEXA peaks, previously described. Therefore, it is possible to affirm that the hydrophilic and the lipidic components seemed to not influence each other during the lyophilization process that gave rise to the final formulation.

In addition to the lipid mixture prepared 24 h before the analysis, a mix kept at 4 °C for 60 days was also analysed, and the respective thermograms were compared, resulting in an entire overlap (data not shown). Both samples melted at 40 °C, indicating the lipid mixture stability over time.

**Figure 4:**
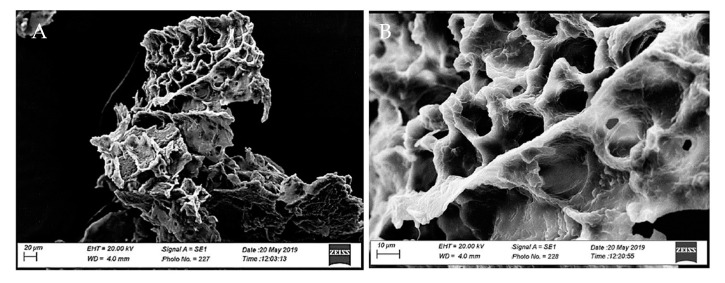
SEM images of surface morphology and internal structure of Sponge-C-MTR. (**A**) magnitude bar = 20 µm; (**B**) magnitude bar = 10 µm.

The structure and the morphology of sponges were investigated. The obtained multicomposite systems were characterized by a porous structure shaped by the water removal during the freeze-drying process, thus generating the vacant spaces, which contributed to the recognized large number of pores. Porosity is the collective term for these pores and their distribution in the structure of the solid. The porosity of a matrix platform influences the ability to adsorb biological fluids as well as release drugs and nutrients embedded in it. The fluid absorption would be cooperative in controlling asepsis at the post-extractive sockets [58]. Moreover, porosity causes an improvement in cell gas exchange, epithelium regeneration, exudates removal, and hemostasis [59,60]. By investigation, the porosity of Sponge-C and Sponge-C-MTR resulted in 93.15 ± 0.14% and 92.86 ± 0.18%, respectively. Of note, that scaffold porosity has been reported as being more than 90% for tissue engineering applications [61]. SEM images, shown in Figure 4, confirmed the porous nature of Sponge-C-MTR. The porous structure was plainly visible, with pores having an average diameter of about 10–20 μm. However, higher resolving power of SEM damaged the sample, thus the presence of NLC trapped in the matrix could not be investigated.

**Figure 5:**
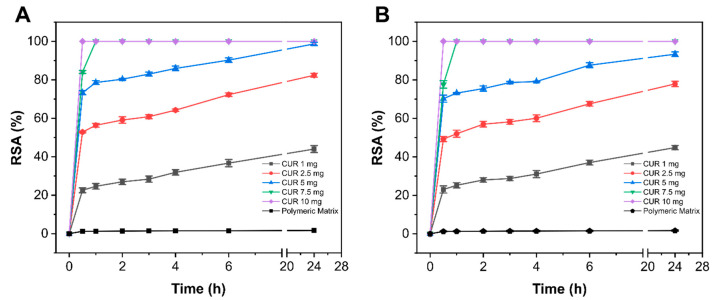
Radical scavenging activity (RSA) of 10, 7.5, 5, 2.5, and 1 mg of Sponge-C (**A**) and Sponge-C-MTR (**B**). The RSA of 10 mg of the Sponge-NLC empty is also reported in the figures and used as a control.

It is well known that ultraviolet and visible light radiations as well as environmental pH are some of the factors involved in the CUR decomposition process. Thus, its incorporation in nanostructured lipid carriers should lead to enhancing stability for maintaining radical scavenging activity. On the other hand, conditions for NLC synthesis, e.g., pH, temperature, or sonication, could cause a loss in antioxidant power due to CUR decomposition. For this reason, the antioxidant activity of Sponge-C and Sponge-C-MTR was evaluated by the ABTS assay. In Figure 5, the results of ABTS assay for the sponges empty and loaded with CUR and CUR-MTR are reported. As expected, the polymeric matrix loaded with empty NLC (named Sponge-NLC empty) did not display considerable antioxidant activity. On the other hand, the presence of CUR within the nanoparticles led to a strong antioxidant effect as a function of the concentration. Indeed, 10 and 7.5 mg of Sponge-C reached 100% of RSA after 30 and 60 min, respectively. Instead, 5 mg of Sponge-C obtained an RSA value of ≈ 99% after 24 h, while 2.5 and 1 mg of Sponge-C showed RSAs of 82% and 44%, respectively. Comparable values and trends of RSA were also found for the Sponge-C-MTR samples. These results suggest the excellent efficacy of Sponge-C and Sponge-C-MTR as an antioxidant platform for the scavenging of harmful free radicals.

**Figure 6:**
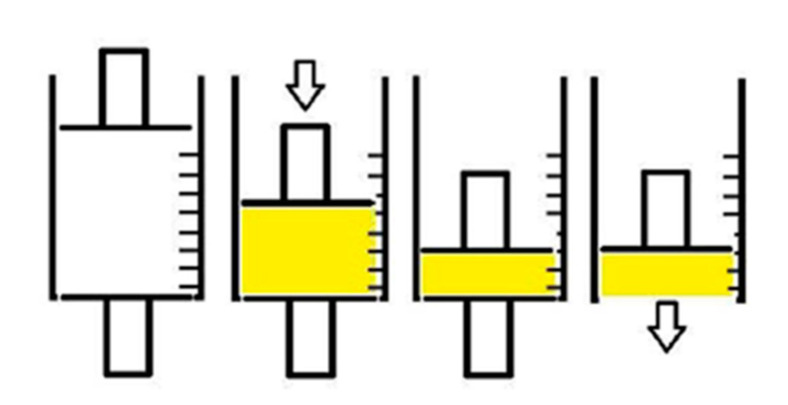
Schematic representation of the process to obtain the minitablets.

Afterwards, minitablets were obtained by compacting definite amounts of Sponge-C-MTR until a definite volume (Figure 6) and were designed to give to the formulation suitable form and dimension for the post-extraction socket to standardize the drug administration dose and to be easily placed into the target size.

The reproducibility of the preparation method was confirmed by measuring average tablet weight, diameter, and thickness, which resulted in 30 ± 1.2 mg, 4.5 ± 0.05 mm, and 2.5 ± 0.05 mm, respectively, and by assessing the data following the Italian Pharmacopoeia [F.U. XII ed.] requirements. Consequently, minitablets from Sponge-C-MTR were used for the next analyses.

Swelling tests as weight and visual assessments were performed to evaluate the morphological changes that may occur in the tablets once in contact with the post-extraction cavity fluids as well as water penetration and sponge hydration ability.

The maximum swelling degree was reached after 15 min, corresponding to a 2.7-fold increase of the initial weight, due to saliva absorption by minitablet forming a viscous gel layer around it.

**Figure 7:**
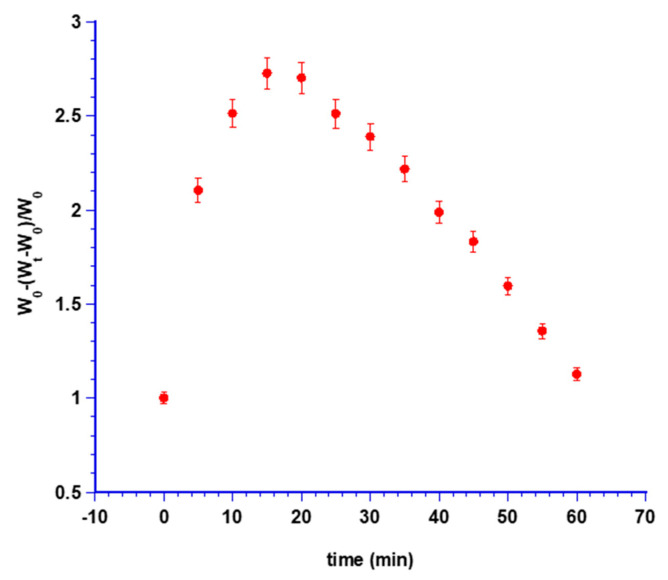
Swelling index measured as weight increase versus time.

Figure 7 shows minitablet weight variations expressed as the increase in the initial weight versus time.

Afterward, the weight decreased due to erosion phenomena of the gel layer and dissolution of MTR together with hydrophilic sponge components that were removed together with the excess of artificial saliva during the experiment.

**Figure 8:**
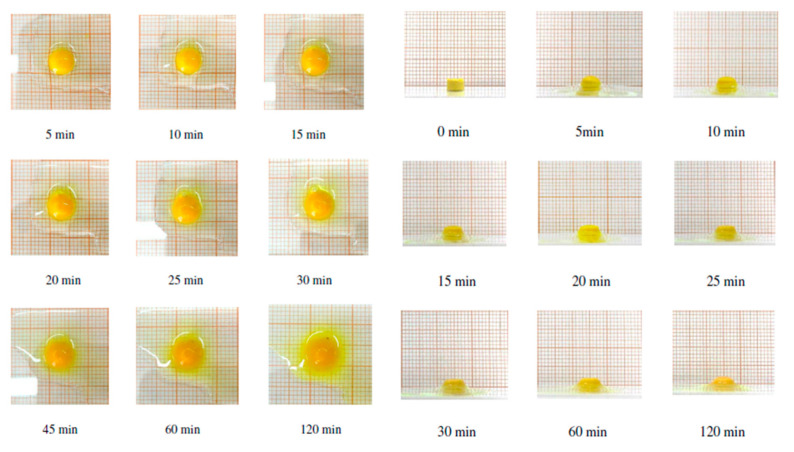
Timeline from 0 to 120 min of radial and frontal swelling of the minitablet.

The swelling aptitude of the minitablets was also evaluated by frontal and radial visual assessments (Figure 8) to exclude any discomfort due to the morphologic changes and to ensure good patient compliance.

In both assessments, a partial loss of weight and dissolution occurred in 120 min, showing a low aptitude of the minitablets to swell.

**Figure 9:**
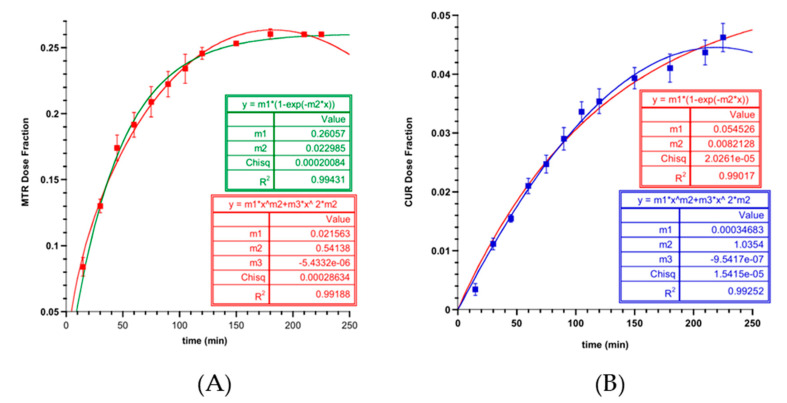
Release data of MTR (**A**) and CUR (**B**) from minitablet, approximated with Peppas–Sahlin and first order mathematical models. Values are presented as means ± SE (*n* = 6).

The minitablets’ ability to release the drugs entrapped respectively in the NLC and in the dried matrix network was evaluated by performing the releasing tests in strict accordance with the different aptitudes and characteristics of compounds. Thus, isopropyl myristate or citrate buffer enriched in 4% (w/v) Polisorbate20 were chosen as acceptor fluids to observe the CUR or the MTR release individually, since there is not a common medium in which both drugs are soluble. The results of release experiments, expressed as dose fraction released versus time (Figure 9), suggest that several physicochemical phenomena are involved in the release mechanisms of the drugs. In CUR release, a lag time was shown that might have been due to the required initial uptake of fluids followed by the sponge swelling that leads to the NLCs release. Instead, the MTR, being trapped in the hydrophilic network, was released and promptly moved by the concentration gradient.

In addition, it is crucial to understand the mechanism by which drugs are released, having developed a complex nanocomposite wherein CUR and MTR are incorporated in lipid nanoparticles and in a hydrophilic matrix, respectively. For this purpose, the most common mathematical models used in dissolution analysis were curve fitted to the experimental data, performing the regression analysis [62]. Mathematical models considered, such as zero- and first-order, Higuchi, Korsmeyer–Peppas, and Peppas–Sahlin, enable the quantitative interpretation of the values obtained from drug release assay [63]. Fitting analyses are summarised in Table 5.

The Peppas–Sahlin model is based on diffusion (Fickian transport) and relaxation due to state-transition in hydrophilic glassy polymers, which swell in water or biological fluids (Transport Case II). This model attempts to quantify the relative contributions of two mechanisms involving dosage form dissolution and introduces two constants that reflect the relative contribution of each mechanism, allowing the quantification of each parameter. The results indicate that the release of the active agents from the system is governed by the combination of Fickian diffusion and Case II relaxation.

However, as reported in Table 5, it was observed that the first-order equation that considers the maximum fraction of a drug released during the process as less than one also fits well with the MTR data.

Therefore, it is possible to consider that the process of release of drugs is governed mainly by a diffusive mechanism highlighted by the low value of Fmax but is limited by their solubility in an aqueous medium. When in contact with biological fluids, MTR, trapped in the hydrophilic polymer chains, forms a saturated solution that drives the diffusion in the acceptor compartment. This outcome was also found for the CUR, although its solubility in physiological fluids was even lower. In fact, CUR, trapped in NLC and surrounded by aqueous medium gradually reached the lipophilic acceptor compartment where it distributed.

These phenomena were probably due to the release evaluation system adopted (Franz cell), which uses a small volume of liquid in the donor compartment (0.5 mL of artificial saliva) in stagnant conditions that produces a saturated drug solution. However, these stagnant conditions are similar to those in which the minitablet can find the post-extraction socket.

After 2 h, the MTR and the CUR released from the formulation resulted in 25% and 4% of the dose, respectively, showing a slow and sustained release of drugs over time, which lead to their prolonged effects.

Recently, non-transfusional hemocomponents have been more and more applied in several dental and oral surgeries procedures, such as the preservation of bone regeneration in post-extraction sockets [64].

The formulation was designed to be administrated in the target site wrapped in the L-PRF membrane, enhancing its well-known regenerative properties with the synergic action given by the simultaneous prolonged delivery of antibiotic and antioxidant compounds.

The ability of the minitablet to promote CUR and MTR permeation rather than penetration in the L-PRF clot was evaluated in agreement with FDA and CE approved protocol (Intra-spin^®^, Intra-lock, Salerno, Italy). An L-PRF clot was mounted as a membrane in Franz type diffusion cells, and the minitablet was applied to the apical side. At the end of each experiment, the drugs entrapped in the L-PRF membrane were also quantified.

**Figure 10:**
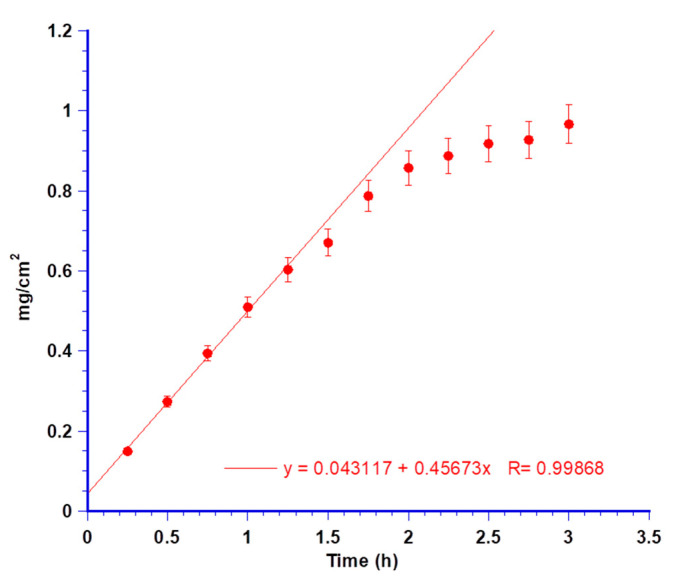
Permeation data of MTR per cm2 throughout the leukocyte and platelet-rich fibrin (L-PRF) membrane and linear fitting at the steady-state concentrations. Values are presented as means ± SE (*n* = 6).

In Figure 10 are the results of MTR amounts permeated in the acceptor chamber versus time. An increased permeation of the antibiotic was noticed, demonstrating its ability to cross the L-PRF membrane. In contrast, it was not possible to detect CUR in the same compartment, probably due to its poor solubility in the acceptor fluid and high affinity with the membrane where it remained entrapped. The extrapolated flux (Js) per unit area of MTR through the L-PRF membrane resulted in 0.457 mg/cm^2^h. Thus, L-PRF acted as the limiting factor for the free diffusion of MTR from the minitablet towards the post-extraction socket. Due to the modulation over time of the antibiotic release and the spreading in the neighbour gingival tissues, the aseptic conditions might have been maintained for a prolonged time in the areas involved in tissue regeneration.

**Figure 11:**
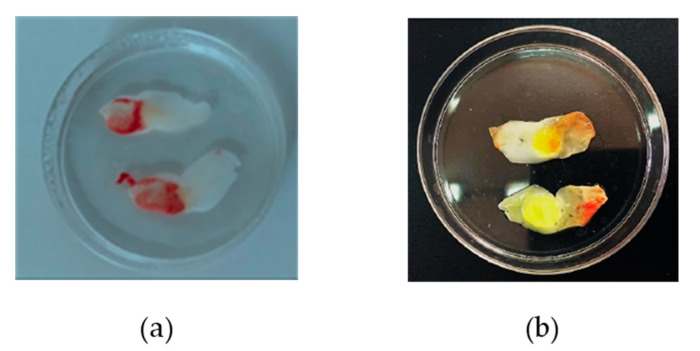
L-PRF membranes before (**a**) and after (**b**) permeation tests.

The amounts of CUR and MTR accumulated in the L-PRF membrane at the end of each experiment (Figure 11b) were evaluated by methanol extraction.

The extracted CUR and MTR, expressed as a percent of the dose administered, resulted in 1.76% w/w and 2.5% w/w, respectively. The accumulation of the drugs in the membrane could validate that the new nanocomposite system acts as a drug reservoir able to release the actives in the surrounding tissues slowly.

The permeation and the accumulation findings, together with release results, were strictly in accordance with our aim of obtaining a formulation able to slowly release anti-inflammatory and antibiotics agents, providing them in the target site for few days.

Based on the obtained data, CUR and MTR abilities to reach the systemic circulation or accumulate in the post-extraction tissues were investigated. The porcine buccal mucosa was used as the membrane model, since its composition is considered the most representative of the human tissues for morphology, lipid content, thickness, and lack of keratinization [65].

**Figure 12:**
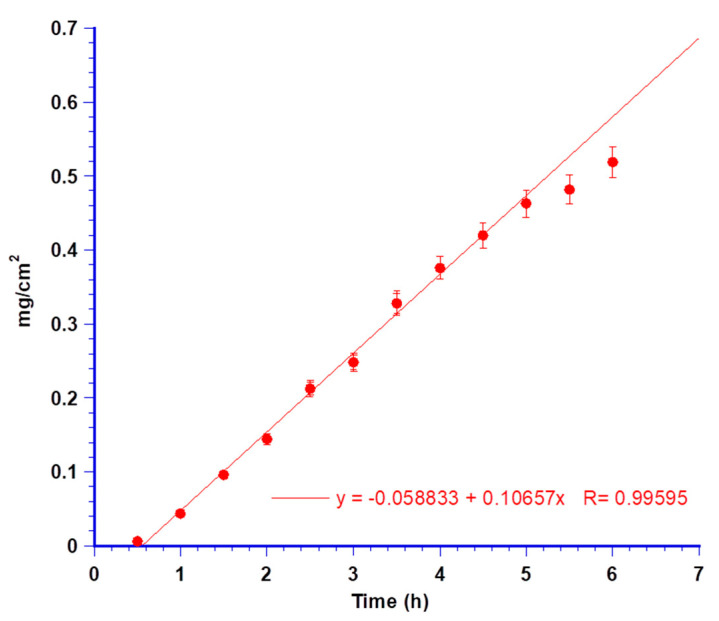
Permeation data of MTR per cm^2^ throughout porcine buccal mucosa and linear fitting at the *steady-state* concentrations. Values are presented as means ± SE (*n* = 6).

Again, MTR was the only drug detected in the acceptor compartment. Plotting the permeated amount per unit area versus time (Figure 12), the obtained flux (Js) resulted in 0.106 mg/cm^2^ h.

At the end of each experiment, the accumulated MTR and CUR inside the mucosa were quantified by methanol extraction, resulting in 10.8% and 17.45% of the applied dose, respectively.

**Figure 13:**
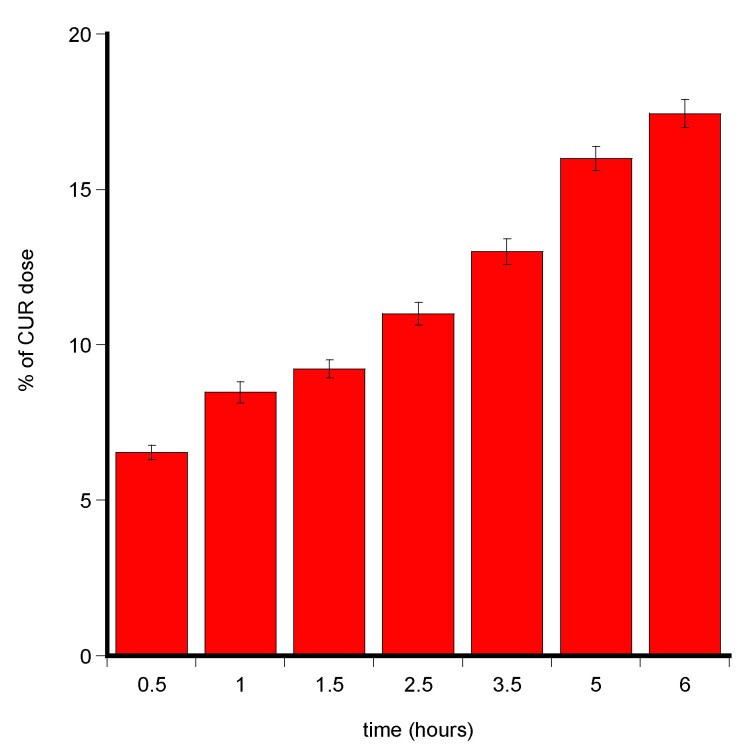
Percent of CUR dose recovery from the porcine membrane at different time points. Values are presented as means ± SE (*n* = 3).

CUR absence in the acceptor fluid and its detection in both L-PRF and porcine mucosa membranes further highlighted the affinity of CUR for lipid components of tissues rather than a propensity to reach the bloodstream and the ability of NLC to promote CUR partitioning in them. The aptitude of CUR to accumulate in the buccal membrane over time was investigated. Further permeation experiments were carried out by ending at different time points (from 30 to 360 min) to withdraw and analyse the porcine membrane. The results are shown in Figure 13 as a percent of CUR dose entrapped in the membrane *versus* time.

CUR amount accumulated in the buccal membrane after the minitablet administration progressively increased, indicating the high affinity of CUR towards biological membrane.

These latest results defined NLCs ability to promote the CUR penetration in the lipophilic domains of the mucous membranes where it was accumulated, carrying out its therapeutic activities as an adjuvant in tissue regeneration. Moreover, MTR was able to penetrate the mucosal tissue, performing its loco-regional and systemic antibacterial activities.

In order to exclude any cytotoxic effects induced by the formulation, the in vitro viability assay on normal stromal cells HS5 was performed by testing Sponge-C-MTR and comparing the results to empty sponge prepared by the same process but without CUR and MTR.

**Figure 14:**
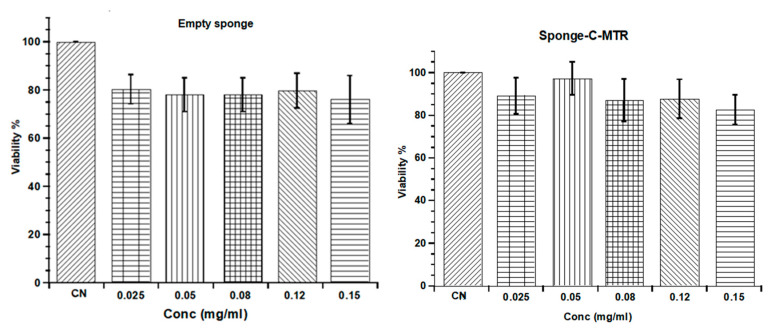
Cell viability of homo sapiens bone marrow stromal cells (HS5) cells after 24 h of treatment at different concentrations of the empty nanocomposite sponge or the Sponge-C-MTR compared to the cell viability of untreated cells (CN). Results were the means ± SE of three separate experiments.

The influence of Sponge-C-MTR on cell viability was investigated by performing the MTT assay after 24 h incubation using a concentration range of 0.025–0.15 mg/mL. According to the results, shown in Figure 14, cell viability was almost not affected by the Sponge-C-MTR, resulting in 80–90% referred to untreated control cells for the whole concentration range analysed. On the other hand, the treatment with any concentration of the empty sponge led to viability between 80–70% with respect to the untreated control, indicating a borderline cytocompatibility. Therefore, the comparison between these systems allowed defining the cytoprotective roles of CUR and MTR.

Although CUR is well known for the anti-cancer activity, this compound shows a biphasic effect on the stem cell proliferation, depending on the treatment concentrations and the type of stem cells used. It has been reported that bone marrow mesenchymal stem cells showed an increase in proliferation at CUR concentration up to 10 µM, whereas they showed a cytotoxic effect for higher concentrations [66]. In our experiments, the tested concentrations of CUR ranging from 0.61 and 3.66 µM were lower than the cytotoxic condition, thus suggesting a protective effect of CUR on cells and making Sponge-C-MTR potentially useful for in vivo administration.

## 4. Conclusions

A multifunctional bioerodible nanocomposite containing metronidazole and curcumin NLCs was designed to be applied together with the L-PRF^TM^ clot, a hemocomponent widely used in oral and dental surgery, to promote bone and tissue regeneration in the post-extraction socket. In this work, the presented drug delivery system was successfully developed and characterized. Sustained releases of CUR and MTR were entrapped in the NLCs, and the bioerodible matrix network of hyaluronic acid was described. The CUR penetration and the MTR permeation throughout both the L-PRF clot and the buccal mucosa were demonstrated, thus entailing the sustained antibacterial, antioxidant, and anti-inflammatory effects in the target site. The biocompatibility of the nanocomposite was also proven.

In light of the described results, we can conclude that this synergistic effect can be a potential strategy to overcome the antibiotic resistance mechanism that may occur with the MTR oral administration and improve the CUR delivery, supporting and enhancing the role of hemocomponents for tissue regeneration in dental and oral fields.

## Figures and Tables

**Table 1:** Composition of lipid mixtures.

SAMPLE	CUR (g)	GA (g)	HEXA (g)	IP (g)	HEXA: IP	Appearance
MIX1	0.08	0.10	0.49	0.33	60:40	Opalescent
MIX2	0.08	0.10	0.41	0.41	50:50	Opalescent
Mix3	0.05	0.10	0.51	0.34	60:40	Opalescent
MIX4	0.05	0.10	0.425	0.425	50:50	Opalescent
Mix5	0.08	0.03	0.53	0.36	60:40	Opalescent
MIX6	0.08	0.03	0.445	0.445	50:50	Opalescent
Mix7	0.05	0.03	0.55	0.37	60:40	Clear

CUR: curcumin; GA: glycyrrhetic acid; HEXA: 1-hexadecanol; IP: isopropyl palmitate.

**Table 2:** Composition of prepared nanostructured lipid carriers (NLCs) using 20 mL of aqueous medium.

Sample	Amount of Mix7 (mg)	Tw80 (mg)	F-68 (mg)
M7-100-T	100	200	-
M7-200-T	200	200	-
M7-300-T	300	200	-
M7-100-TP	100	200	200
M7-200-TP	200	200	200
M7-300-TP	300	200	200

**Table 3:** Mean particle size (Z-average), particle size distribution (PDI), and electrical charge surface (ZP) of NLC formulations. Values are expressed as mean ± SD (*n* = 3).

Samples	Z-Average (nm)	PDI	Z-Potential (mV)
M7-100-T	121.8± 15.44	0.372	−20.5 ± 6.8
M7-100-TP	117.9 ± 32.81	0.290	−19.5 ± 5.1
M7-200-T	88.5 ± 8.68	0.733	−19.5 ± 6.44
M7-200-TP	182.2 ± 36.88	0.265	−13.5 ± 7.95
M7-300-T	689.4 ± 17.08	0.709	−20.5 ± 5.87
M7-300-TP	157.0 ± 25.52	0.403	−25.5 ± 7.79
M7-300-TP-MTR	112.0 ± 28.56	0.337	−24.0 ± 5.32

**Table 4:** Nanocomposite sponge compositions.

	Composition of Each Batch of Composite Sponges (mg)							
	CUR-NLC	MTR	HyNa	PVP-K90	TRH	Tw80	P-68	Buffer Salts
Sponge-A	M7-300-TP	0	50	8	300	200	200	57
Sponge-A-MTR	M7-300-TP-MTR	150	50	8	300	200	200	57
Sponge-B	M7-300-TP	0	100	16	600	200	200	57
Sponge-B-MTR	M7-300-TP-MTR	150	100	16	600	200	200	57
Sponge-C	M7-300-TP	0	160	24	400	200	200	57
Sponge-C-MTR	M7-300-TP-MTR	150	160	24	400	200	200	57

**Table 5:** Analysis of kinetic model parameters values for MTR and CUR released from minitablet.

	MTR				CUR			
Parameters	k	*n* or m	r2	c2	k	*n* or m	r2	c2
$F=k_{0}\times t$ Zero-order	1.60 × 10^−3^		too low	6.78 × 10^−2^	2.42 × 10^−4^		0.798	4.15 × 10^−4^
$F=100\times \left(1-e^{-k_{1}\times t}\right)$ First-order ^a^	1.60 × 10^−5^		too low	6.76 × 10^−2^	2.43 × 10^−6^		0.799	4.15 × 10^−4^
$F=F_{max}\times \left(1-e^{-k_{1}\times t}\right)$ First-order with Fmax ^b^	2.30 × 10^−2^	Fmax = 0.261	0.994	2.01 × 10^−4^	8.21 × 10^−3^	Fmax = 0.055	0.990	2.03 × 10^−5^
$F=k_{H}\times t^{0.5}$ Higuchi ^c^	2.065 × 10^−2^		0.735	9.32 × 10^−3^	3.00 × 10^−3^		0.929	1.47 × 10^−4^
$F=k_{KP}\times t^{n}$ Korsmeyer–Peppas ^d^	4.77 × 10^−2^	0.32788	0.916	2.96 × 10^−3^	1.54 × 10^−3^	0.6355	0.961	8.01 × 10^−5^
$F=k_{1}\times t^{m}+k_{2}\times t^{2m}$ Peppas–Sahlin ^e^	k1= 2.16 × 10^−2^ k2= −5.43 × 10^−6^	0.54138	0.992	2.86 × 10^−4^	k1 = 3.47 × 10^−4^ k2 = −9.54 × 10^−7^	1.0354	0.993	1.54 × 10^−5^

In all models, F is the fraction of drug released in time t. ^a^ k_1_ = first-order release constant, ^b^ Fmax = maximum fraction of the drug released at infinite time, ^c^ k_H_ = Higuchi release constant, ^d^ k_KP_ = release constant incorporating structural and geometric characteristics of the drug-dosage form; *n* is the diffusional exponent indicating the drug-release mechanism, ^e^ k_1_ is the constant related to the Fickian kinetics; k_2_ is the constant related to Case-II relaxation kinetics; m is the diffusional exponent for a device of any geometric shape which inhibits controlled release. Plotting the fraction released (Mt/M∞) on time (min), the best fitting with the experimental data of CUR and MTR coupled was obtained by applying the Peppas–Sahlin equation, as shown in Figure 9.
